# The Use of MiniMed780G System Is Associated With Stable Glycemic Control in People With Type 1 Diabetes Before, During, and After Ramadan: An Observational Study

**DOI:** 10.1155/jdr/4144787

**Published:** 2025-01-02

**Authors:** Abdullah M. Alguwaihes, Najla Alotaibi, Metib Alotaibi, Naglaa Masry, Saher Safarini

**Affiliations:** ^1^Endocrinology Unit, Internal Medicine Department, College of Medicine, King Saud University, Riyadh, Saudi Arabia; ^2^Diabetes Center, Dallah Hospital, Riyadh, Saudi Arabia; ^3^Internal Medicine, King Saud University Medical City, Riyadh, Saudi Arabia; ^4^University Diabetes Center, King Saud University Medical City, Riyadh, Saudi Arabia

**Keywords:** advanced hybrid closed-loop system, fasting, insulin pump, Ramadan, Type 1 diabetes

## Abstract

**Aims:** The study was aimed at assessing the role of the MiniMed780G system of glycemic control before, during, and after Ramadan among people with Type 1 diabetes (PwT1D).

**Methods:** This is a single-center retrospective analysis of MiniMed780G system users aged 14 years and above whose glycemic profiles were collected from February 21 to May 20, 2023, which corresponds to the Hijri months of Sha'ban, Ramadan, and Shawwal 1444/1445. Data was collected, processed, and analyzed in the framework of the Medtronic Galaxy service of the One Hospital Clinical Service (OHCS) program in Dallah Hospital, Riyadh, Saudi Arabia. Data from 43 PwT1D (24 females, mean age 30 ± 11 years with 14 ± 8 years from diabetes onset) using the MiniMed780G system were collected.

**Results:** Overall, the 3-month (Sha'ban, Ramadan, and Shawwal) mean sensor glucose (SG), time in range (TIR) (70–180 mg/dL), time below range (TBR) (54–69 mg/dL and < 54 mg/dL), time above range (TAR) (180–250 mg/dL and > 250 mg/dL), and glucose management indicator (GMI) showed no statistical differences within the three periods. No differences in insulin total daily dose have been detected, and no diabetic ketoacidosis (DKA) or severe hypoglycemia events occurred.

**Conclusion:** The use of the MiniMed780G system is safe with favorable glycemic outcomes across nonfasting and fasting months.

## 1. Introduction

Type 1 diabetes (T1D) is a chronic autoimmune condition in which the pancreas does not produce insulin. As of 2021, more than 8.4 million individuals globally have T1D, and this is projected to almost double in 2040, reaching 13.5–17.4 million [1]. While T1D was previously known as juvenile diabetes, the recent global epidemiologic evidence actually revealed that more than half (64%) of the newly diagnosed people with Type 1 diabetes (PwT1D) were adults 20–59 years old, and almost a fifth (19%) were 60 years and above [1]. This apparent shift in T1D demographics may have little influence on the overall management of the disease since T1D outcomes and characteristics in both children and adults have little distinction, and subtle differences (e.g., the incidence of diabetic ketoacidosis (DKA) being more common in children and lower use of insulin pumps in adults) imply that achieving glycemic control remains consistently challenging, regardless of the age group [2]. Increased risk of serious complications such as DKA is triggered mostly by stronger external factors such as infection and poor compliance [3].

Blood glucose monitoring remains one of the cornerstones of T1D management, as it is the basis for controlling blood glucose levels via the provision of insulin, coupled with a healthy diet and lifestyle to prevent complications [4]. The overall treatment, however, is geared only towards control, not cure. Hence, PwT1D carries a lifetime condition whose survival is heavily tied to a lifelong adherence to an insulin regimen. Factors such as cultural and religious obligations can severely test the adherence to PwT1D, making a seemingly straightforward management complicated. Among these religious factors is the obligatory fasting during the Hijri month of Ramadan, the ninth lunar month of the Islamic calendar [5].

Fasting during the holy month of Ramadan is one of Islam's five pillars of faith, and ~43% of Muslims with T1D fast during Ramadan, the lowest being 9% in Morocco and the highest 72% in Saudi Arabia [5], according to (or against) religious guidance and medical advice [6]. Fasting consists of complete abstinence from eating, drinking (including water), and medication from dawn to sunset [7, 8]. While theoretically a month-long fast during Ramadan may expose PwT1D to an increased risk of experiencing episodes of hypoglycemia, hyperglycemia, or DKA, real-time evidence has been largely inconsistent [9–12]. This discrepancy is mostly because the populations used for observational studies have varying levels of baseline glycemic status, indicating that risk stratification is pivotal as to which type of population will benefit the most from increased glucose monitoring during seasonal religious practices such as Ramadan. Hence, and given the severity of the risks involved, management of diabetes independent of type during Ramadan and proper fasting guidance remain critical [13]. Fortunately, within the last two decades, the management of diabetes and T1D in particular, independent of seasonal lifestyle changes, was revolutionized with the introduction of continuous glucose monitoring (CGM) devices [14].

CGM devices have been increasingly adopted by PwT1D, as standalone devices or as part of integrated devices with insulin delivery devices, in both open and closed-loop modes. The MiniMed780G advanced hybrid closed-loop (AHCL) system has been successful in achieving and exceeding international glucose targets in various populations, ages, and background therapies and in different clinical settings, including Ramadan [15–20]. A feature of the MiniMed780G system is the revolutionary SmartGuard algorithm, which, depending on the real-time sensor glucose (SG) level, delivers insulin automatically to prevent abnormal glucose level fluctuations, adapting to the individual characteristics, all of which occur with no interference from the patient [21].

Given the accumulating data on the efficacy of integrated CGM devices with insulin pumps such as the MiniMed780G, it can be hypothesized that minimal difference should be observed over time in terms of achieving glycemic control among PwT1D who fast during Ramadan. Hence, the present retrospective study was aimed at assessing the efficiency of the MiniMed780G AHCL system by comparing the glycemic control achieved by PwT1D before, during, and after Ramadan.

## 2. Materials and Methods

### 2.1. Study Design and Population

This is a single-center retrospective study conducted at the Diabetes and Endocrinology Center of Dallah Hospital, a private tertiary hospital in Riyadh, Saudi Arabia. Saudi male and female patients aged 14 years and above who are known PwT1D attending the outpatient clinics of the Diabetes and Endocrinology Center and participating under the framework of the One Hospital Clinical Service (OHCS) project of Medtronic were screened for inclusion. These participants are active users of the Medtronic MiniMed780G AHCL system (Medtronic, Northridge, CA, United States) during the nonfasting month before Ramadan (Sha'ban—February 21, 2023, to March 22, 2023), Ramadan (March 23, 2023, to April 20, 2023), and again the nonfasting month after Ramadan (Shawwal—April 21, 2023, to May 20, 2023). Inclusion in the final analysis required that the patient achieved 70% of the time in SmartGuard automation and had ≥ 70% of the time with sensor data. All patients were on insulin aspart (NovoRapid, Novo Nordisk, Copenhagen, Denmark). Ethical approval and waiver of consent were obtained from the Institutional Review Board (IRB) of Dallah Hospital, Riyadh, Saudi Arabia (IRB No.: E-22-6980).

### 2.2. Data Collection of Glycemic Indices and Study Endpoints

Saudi PwT1D was retrospectively followed by Dallah Hospital under the framework of the Medtronic Galaxy service—part of OHCS, a medical care program provided by Medtronic. This service aggregates data from different sources (clinical, device, and patient data) and generates outcome data for research, benchmarking, and across-site collaboration. In brief, the Medtronic Galaxy service of the OHCS provided technical and statistical support for the collection, management, analysis, and reporting of pseudonymized data of the institution's patients treated by or indicated to Medtronic devices, therapies, or solutions. These data and related information were provided back to the institution to improve knowledge about applied therapies and optimize patients' outcomes. As part of the OHCS, the collaborating institution can manage clinical and diagnostic data (for devices that allow diagnostic data storage) collected during hospital visits, visits carried out through remote connections, or other conditions according to the institution's clinical practice. Medtronic provides case report forms to the institution to collect clinical data through the OHCS data collection platform, managed by a certified external service provider. The institution receives access to the website from Medtronic for data management, periodic reports, and ad hoc statistical analyses.

Glycemic and insulin delivery data were collected through regular uploads, which can be done automatically (device/system initiated) or manually (carbohydrate-based or patient's attempt to correct) according to the user's choice. The data retrieved from the software for the purpose of the present analysis included the SG, percentage of time in target (time below range (TBR), time in range (TIR), and time above range (TAR)), glucose management indicator (GMI), standard deviation (SD), coefficient of variation (CV), average total daily insulin dose, average daily (percent) of autocorrection bolus insulin, average daily (percent) manual bolus insulin, sensor use (percent), and time in auto mode (percent).

### 2.3. Data Analysis

Descriptive statistics were used to summarize all results. These include mean and SD for continuous variables and frequencies (percent) for categorical variables. Mean changes in the outcomes of interest over the three periods (Sha'ban, Ramadan, and Shawwal) were estimated using linear mixed models to account for the within-patient correlation. Estimates and their 95% Confidence Intervals (CIs) were provided. Comparisons between Ramadan fasting and nonfasting hours (5 AM–6 PM) were also performed using the Wilcoxon signed-rank test. Glucose profiles for the different months were plotted. All statistical tests were based on a two-sided significance level of 0.05. No adjustment was made for any multiple comparisons. SAS software, Version 9.4, (SAS Institute Inc., Cary, NC, United States) was used to perform statistical analyses.

## 3. Results

**Figure 1:**
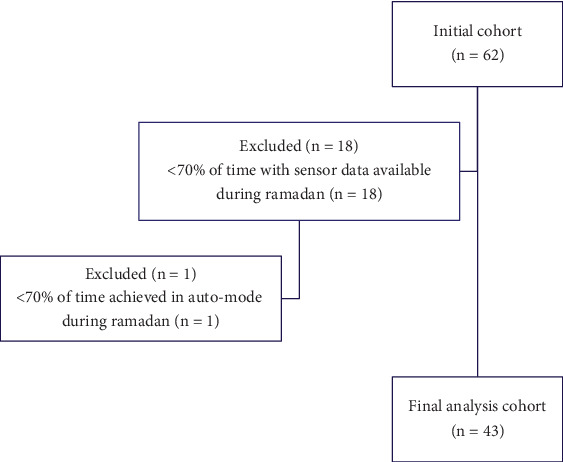
Flowchart of participants.

A total of 62 PwT1D records were screened, out of which 19 were excluded for failure to achieve the % time in SmartGuard automation and sensor data in the 3-month study period required for final analysis, bringing the final sample size to *N* = 43 (see Figure 1).  Table 1 shows the demographic characteristics of the analyzed patients. Mean age at baseline was 30.0 years (ranging from 14.0 to 64.0), and more than half of the patients were females (56%). Five (12%) patients had thyroiditis, and another five (12%) had hypertension. Regarding medical history, only one (2%) patient had celiac disease. Chronic complications were reported by eight (19%) patients, five (12%) were affected by diabetic nephropathy, four (9%) by diabetic retinopathy, and three (7%) by neuropathy (7%). None of the participants had cerebrovascular and cardiovascular diseases (Table 1). Glucose profiles for the month of Ramadan were plotted and compared to other months and showed two distinct spikes at the hours of iftar and suhur (Figure S1), confirming that most of the patients were fasting.

**Figure 2:**
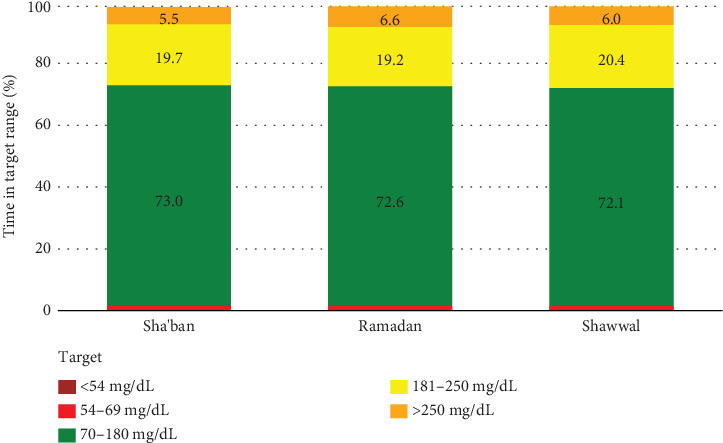
Mean percentage of time in target by period.

**Figure 3:**
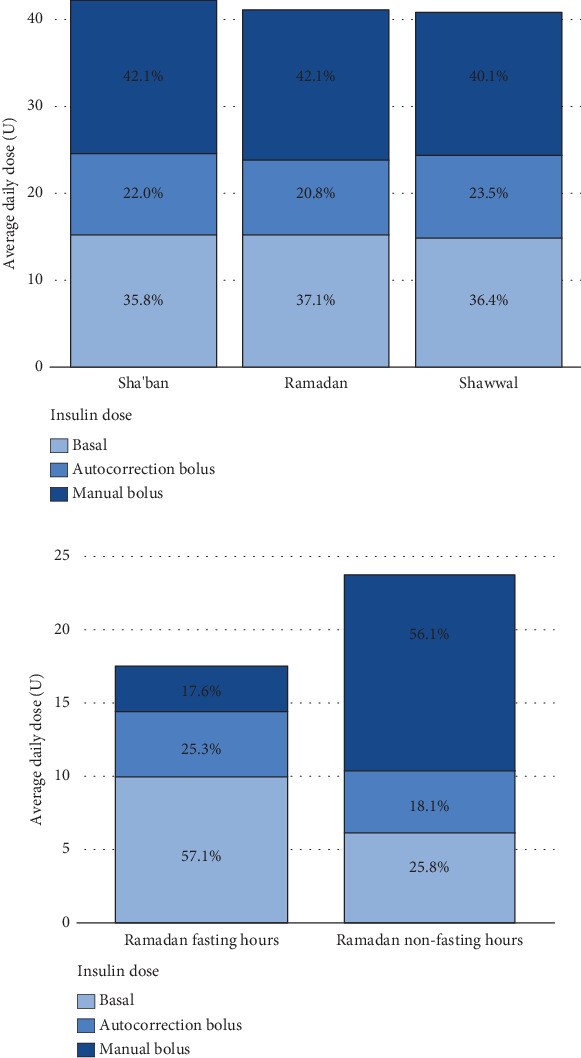
(a) Average daily insulin dose and (b) insulin dose according to fasting and nonfasting hours.

Table 2 shows the glycemic variability, TIR, and mode usage over time. No significant differences were observed over time in all SG indices, including SD and CV. GMI was also comparable across the three Hijri months. The same nonsignificance was observed when results obtained were compared between Ramadan versus Sha'ban, Shawwal versus Sha'ban, and Shawwal versus Ramadan. Furthermore, no significant differences were seen in TIR indices over time and when compared between Ramadan versus Sha'ban, Shawwal versus Sha'ban, and Shawwal versus Ramadan (Figure 2). In terms of sensor use, the mean percentage was lowest during the month of Shawwal (89.8 ± 4.5), and the mean changes were significantly lower compared to both Sha'ban (−3.7; *p* < 0.001) and Ramadan (−5.2; *p* < 0.001). Mean SmartGuard automation percentage during the month of Shawwal (92.9 ± 5.5) was also the lowest compared to both months, with mean changes significantly lower than Sha'ban (−2.2; *p* = 0.02) and Ramadan (−3.8; *p* < 0.001). Lastly, mean changes in the daily insulin doses were significantly lower during Shawwal than during Sha'ban (−3.0; *p* = 0.01). No difference in mean insulin changes was seen when Shawwal and Ramadan were compared. Lastly, no significant differences were observed in percentage basal, autocorrection, and manual insulin over time (Table 2 and Figure 3(a)).

The same parameters were compared between fasting and nonfasting hours (Table 3) and showed that indices of glycemic variability including SG mean, SG SD, SG CV%, and GMI% were significantly (*p* < 0.05) increased during nonfasting hours than fasting hours. TIR 70–180 mg/dL was significantly higher during the fasting hours (*p* < 0.001), while TAR 181–250 mg/dL and TAR > 250 mg/dL were significantly reduced in the fasting hours (*p* < 0.001). Lastly, the insulin requirement by PwT1D during fasting and nonfasting hours differed significantly (Figure 3(b)), with both mean basal and autocorrection bolus (percent) values observed to be significantly lower during fasting than nonfasting hours (*p* values < 0.001). Nonfasting hours, on the other hand, had significantly higher insulin units and manual bolus (percent) than fasting hours (*p* < 0.001).

**Figure 4:**
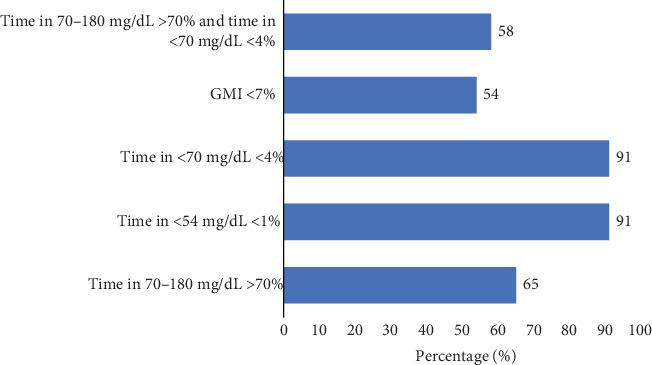
Achievements of consensus glucose targets during Ramadan.

Figure 4 shows the overall prevalence of PwT1D achieving the consensus glucose targets during Ramadan, with time in 70–180 mg/dL > 70% met by 65% (*N* = 28) of participants, time in < 54 mg/dL < 1% and time in < 70 mg/dL < 4% achieved by 91% (*N* = 39) of participants, GMI < 7% by 54% (*N* = 23) of participants, and combined time in 70–180 mg/dL > 70% and < 70 mg/< 4% by 54% (*N* = 25) of participants. Lastly, no common patterns were observed in terms of device settings (Table S1), and no significant changes in device settings were observed over time (Table S2). The distribution of patients according to configuration (i.e., a combination of glucose target and Active Insuline Time (AIT)) used for more than 95% of the time, if present, is shown in Figure S2. Finally, no adverse effects such as skin reactions due to adhesives used in sensors were noted, as well as complications including hypoglycemia and DKA during the study duration.

## 4. Discussion

The present study assessed the efficacy of Medtronic MiniMed780G among PwT1D in terms of stabilizing glycemic variability from retrospective data obtained from the Medtronic Galaxy service during the Hijri months of Sha'ban, Ramadan, and Shawwal. The major findings indicate that the indices of glycemic variability and time in the target were comparable across the three lunar months, and no significant changes were observed when comparing values obtained during Ramadan versus Sha'ban, Shawwal versus Sha'ban, and Shawwal versus Ramadan. Furthermore, no significant changes were observed when the data were compared across the 3 months of observation with respect to the delivered insulin. The results specifically highlight that variations before and after Ramadan were similar to values during Ramadan, ultimately suggesting that PwT1D on MiniMed780G can safely fast during the holy month without adversely affecting optimum TIR (> 70%) and without increased risk of hypoglycemia. The findings of the present study largely echo previous observations on the efficacy of the MiniMed780G and its ability to swiftly adapt to substantial lifestyle changes [21–26]. It is worthy to note, however, that the majority of the studies ascertaining the efficacy of the MiniMed780G system have focused on the month of Ramadan alone, at least in largely Muslim populations. What the present study adds to the existing literature is that while the MiniMed780G can be deemed effective in maintaining glucose control prior to and after the month of Ramadan, the persistence of this optimum glycemic control transcends before and after this abrupt month-long lifestyle change, as seen by the nonsignificant differences in CGM parameters over time.

Outside the context of Ramadan, the sustained efficacy of the MiniMed780G over time has also been observed in several populations. In a recent prospective study done in Italy, 368 children and adolescents with T1D were observed for outcomes after 1 full year of use of the MiniMed780G, and it was found that substantial improvements in all glycemic indices were observed within 15 days of auto mode use, and these favorable changes, including glycated hemoglobin (HbA1c), persisted for the entire duration of the study, even among participants with low therapeutic engagement [27]. Among adults with T1D, one prospective study conducted in Sweden assigned 140 PwT1D with either the Tandem Control-IQ or the MiniMed780G and observed the safety of both systems for an average of 1.7 years [28]. The findings revealed that both AHCL systems not only improved glucose control in terms of increased TIR from 57% to 71% and decreased TBR from 3.8% to 1.6% but also increased patient satisfaction [28]. It is worthy to note that in the same study, one-third of participants experienced skin reactions from both treatment groups and that three out of the four cases of severe hypoglycemia were from the MiniMed780G group, and this was due to self-correcting glucose levels, which largely interfered with the autocorrections delivered by the pump [28]. Similar results meeting and sustaining clinical targets using the MiniMed780G for over 12 months were documented in Australia [29]. In contrast, none of these adverse events were observed in the present study. It is worthy to note that at the time of this writing, the majority of the studies mentioned and conducted assessing the efficiency of the AHCL systems were done in developed countries and almost none in low- and middle-income nations where the burden of T1D is relatively unknown [30].

Other findings in the present study were expected responses of the device secondary to the fasting status of PwT1D and the abrupt lifestyle changes during monthly transitions: indices of glycemic variability, including SG mean, SG SD, SG CV%, and GMI%, significantly increased during Ramadan nonfasting hours than fasting hours. TIR 70–180 mg/dL was significantly higher during the fasting hours, while TAR 181–250 mg/dL and TAR > 250 mg/dL were significantly reduced during the fasting hours. These changes were in parallel to the insulin dose responses to not only compensate for the glycemic variabilities induced but also to maintain the optimum TIR, a feature inherent in AHCL devices. Another interesting finding is the relatively elevated CV among participants despite good glycemic control and very low time under and above range, similar to previous studies [16, 23]. Nevertheless, the significance of CV as a marker of glycemic control has been recently found to be poor among MiniMed780G users, with the least contribution in terms of hypoglycemia risk as compared to other indicators. Having a high CV, therefore, amongst users may have little to no clinical value [31].

The authors acknowledge several limitations. First, the retrospective design limits the findings based on available data, and other measures such as patient satisfaction and quality of life were not assessed. These variables, however, were already investigated in other studies having larger scales [28, 32–35]. Second, the lack of adverse events in the present study may be due to the small sample size, as it requires larger cohorts to document these events. Third, the findings need to be interpreted with caution as we obtained these results in PwT1D who were well trained on the use of the technology and well followed-up, and that information, such as the amount of carbohydrates ingested, is still provided by the user and not adjudicated. Furthermore, whether or not the patients or physicians made setting changes based on their intake in any of the periods covered was not recorded to reflect real-world settings and to highlight how the algorithm adapts seamlessly despite the absence of this information. Fourth, the recommended optimal settings, which provide the best outcomes, were used only in a limited number of users, which might lessen the outcomes. Lastly, the study has no control group, which could have been beneficial in data analysis. Despite the caveats, the data collected was robust, and the study was adequately powered, ensuring that the results obtained were reliable, repeatable, and resource-friendly. The study is arguably the first in the Middle Eastern region to perform data collection using the Medtronic Galaxy service of the OHCS program.

## 5. Conclusions

In summary, the use of the MiniMed780G automated insulin delivery system is able to maintain stable glycemic control before, during, and after Ramadan among PwT1D, highlighting the device's adaptability to drastic but temporary lifestyle changes such as Ramadan fasting, which can dramatically alter glycemic control and variability in PwT1D. As the management of T1D continues to evolve in achieving the full “artificial pancreas,” more studies with comparator groups are needed in other T1D populations having diverse cultural, socioeconomic, and psychosocial backgrounds to determine the consistency of the device's safety and efficacy profile in diverse settings and users.

## Figures and Tables

**Table 1:** Demographic characteristics.

*N* (M/F)	43 (19/24)
Age (years)	30 ± 10.8
BMI (kg/m^2^)	26.4 ± 4.7
Duration of T1DM (years)	14.2 ± 8.2
Last HbA1c (%) before Ramadan	7.2 ± 0.7
Medical history and complications (%)	
Celiac disease	1 (2)
Thyroiditis	5 (12)
Hypertension	5 (12)
DM nephropathy	5 (12)
DM retinopathy	4 (9)
Diabetic foot	0
DM neuropathy	3 (7)
Cerebrovascular disease	0
Cardiovascular disease	0

*Note:* Data presented as *N* (percent) for frequencies and mean ± SD for continuous variables.

**Table 2:** Glycemic variability indices, time in ranges, sensor/auto mode usage, and insulin doses during Sha'ban, Ramadan, and Shawwal.

	**Sha'ban**	**Ramadan**	**Shawwal**	**Mean change (95% CI); ** **p** ** value**		
				**Ramadan vs. Sha'ban**	**Shawwal vs. Sha'ban**	**Shawwal vs. Ramadan**
Glycemic indices						
SG mean (mg/dL)	150.8 ± 14.9	152.8 ± 15.5	153.3 ± 16.4	0.3 (−3.2; 3.8); 0.86	0.6 (−2.1; 3.4); 0.64	0.3 (−2.2; 2.9); 0.78
SG SD (mg/dL)	53.5 ± 11.0	56.0 ± 13.2	53.4 ± 11.2	1.0 (−2.1; 4.2); 0.52	−0.5 (−3.0; 2.0); 0.69	−1.5 (−3.8; 0.7); 0.18
SG CV (%)	35.4 ± 5.9	36.4 ± 6.9	34.7 ± 5.6	0.5 (−1.4; 2.4); 0.57	−0.5 (−2.1; 1.0); 0.49	−1.1 (−2.5; 0.3); 0.13
GMI (%)	6.9 ± 0.4	7.0 ± 0.4	7.0 ± 0.4	0.0 (−0.1; 0.1); 0.86	0.0 (−0.1; 0.1); 0.64	0.0 (−0.1; 0.1); 0.78
Time (%)						
< 54 mg/dL	0.4 ± 0.7	0.3 ± 0.4	0.2 ± 0.5	−0.1 (−0.3; 0.1); 0.33	−0.1 (−0.2; 0.1); 0.35	0.0 (−0.1; 0.1); 0.77
54–69 mg/dL	1.5 ± 1.2	1.3 ± 1.1	1.3 ± 1.0	−0.2 (−0.5; 0.2); 0.29	−0.1 (−0.4; 0.2); 0.40	0.1 (−0.2; 0.3); 0.62
70–180 mg/dL	73.0 ± 10.0	72.6 ± 10.1	72.1 ± 10.5	0.5 (−1.9; 3.0); 0.66	−0.2 (−2.1; 1.8); 0.88	−0.7 (−2.4; 1.1); 0.44
181–250 mg/dL	19.7 ± 6.3	19.2 ± 6.3	20.4 ± 6.7	−1.0 (−2.5; 0.6); 0.22	0.0 (−1.2; 1.3); 0.96	1.0 (−0.1; 2.1); 0.08
> 250 mg/dL	5.5 ± 4.9	6.6 ± 5.4	6.0 ± 5.1	0.7 (−0.5; 1.9); 0.24	0.3 (−0.7; 1.3); 0.53	−0.4 (−1.3; 0.5); 0.35
Sensor use (%)	92.7 ± 6.9	93.7 ± 5.2	89.8 ± 4.5	1.5 (−0.1; 3.1); 0.06	−3.7 (−5; −2.5); < 0.001	−5.2 (−6; −4.1); < 0.001
Auto mode (%)	94.6 ± 6.7	96.0 ± 4.3	92.9 ± 5.5	1.7 (−0.5; 3.8); 0.13	−2.2 (−4; −0.4); 0.02	−3.8 (−5.5; −2); < 0.001
Daily insulin doses						
Unit	42.3 ± 18.2	41.2 ± 19.7	40.8 ± 20.6	−2.1 (−5.1; 1.0); 0.18	−3.0 (−5.4; −0.7); 0.01	−1.0 (−3.1; 1.1); 0.36
Basal (%)	35.8 ± 6.9	37.1 ± 6.7	36.4 ± 6.6	1.2 (−1.1; 3.5); 0.31	0.6 (−1.3; 2.5); 0.51	−0.5 (−2.3; 1.2); 0.53
Autocorrection (%)	22.0 ± 10.5	20.8 ± 11.1	23.5 ± 11.1	−1.6 (−4.0; 0.9); 0.20	0.2 (−1.8; 2.1); 0.87	1.7 (−0.0; 3.4); 0.05
Manual (%)	42.1 ± 11.3	42.1 ± 12.6	40.1 ± 12.0	0.4 (−2.3; 3.1); 0.77	−0.8 (−2.9; 1.4); 0.47	−1.2 (−3.1; 0.8); 0.23

*Note:* Significant at *p* < 0.05.

**Table 3:** Glycemic indices and time in target (hours) and distribution of insulin during Ramadan.

	**Nonfasting hours (6 PM–5 AM)**	**Fasting hours (5 AM–6 PM)**	**p** ** value**
Glycemic indices			
SG mean (mg/dL)	167.6 ± 22.8	140.5 ± 13.2	< 0.001
SG SD (mg/dL)	59.5 ± 14.5	47.4 ± 13.7	< 0.001
SG CV (%)	35.5 ± 7.5	33.5 ± 8.1	0.049
GMI (%)	7.3 ± 0.5	6.7 ± 0.3	< 0.001
Time (%)			
< 54 mg/dL	0.3 ± 0.6	0.3 ± 0.5	0.99
54–69 mg/dL	1.5 ± 1.5	1.2 ± 1.1	0.23
70–180 mg/dL	61.2 ± 14.5	82.1 ± 8.9	< 0.001
181–250 mg/dL	26.6 ± 9.2	13.0 ± 5.5	< 0.001
> 250 mg/dL	10.4 ± 8.5	3.4 ± 3.7	< 0.001
Daily insulin doses			
Average daily dose (U)	23.8 ± 11.5	17.5 ± 8.9	< 0.001
Average daily basal insulin dose (%)	25.8 ± 7.2	57.1 ± 10.4	< 0.001
Average daily autocorrection bolus dose (%)	18.1 ± 13.9	25.3 ± 9.0	< 0.001
Average daily manual bolus (%)	56.1 ± 17.1	17.6 ± 11.4	< 0.001

*Note:* Ramadan fasting hours are the dawn to sunset period of complete abstinence from eating, drinking (including water), and medication. Average daily manual bolus (percent) refers to any insulin pumped apart from basal (percent). Average daily autocorrection bolus dose (percent) is the automated correction bolus in the Medtronic device. Data are presented as mean ± SD; significant at *p* < 0.05.

## Data Availability

Data supporting the findings are available within the article.

## Supporting Information

Supporting Information Additional supporting information can be found online in the Supporting Information section. Table S1: Device settings during Sha'ban, Ramadan, and Shawwal. Table S2: Percent changes in device settings. Figure S1: Glucose profiles across months. Figure S2: Device configurations used by the patients (for at least 95% of the time) during Sha'ban, Ramadan, and Shawwal.
