# Supplementary material for: The Use of MiniMed780G System Is Associated With Stable Glycemic Control in People With Type 1 Diabetes Before, During, and After Ramadan: An Observational Study
Source: J Diabetes Res. 2025 Jan 2;2025:4144787. doi: 10.1155/jdr/4144787 (PMC11717436; doi:10.1155/jdr/4144787)
Supplement: Supporting Information — Additional supporting information can be found online in the Supporting Information section. Table S1: Device settings during Sha'ban, Ramadan, and Shawwal. Table S2: Percent changes in device settings. Figure S1: Glucose profiles across months. Figure S2: Device configurations used by the patients (for at least 95% of the time) during Sha'ban, Ramadan, and Shawwal. [file 4144787.f1.docx]

**Supplementary table** **1**. Device settings during Shaban, Ramadan and Shawwal

| **Device Settings** | **Sha'ban** | **Ramadan** | **Shawwal** |
| --- | --- | --- | --- |
| At least 95% of the time with Glucose Target = 100 mg/dL and AIT = 2 hours | 1 (2) | 3 (7) | 3 (7) |
| At least 85% of the time with Glucose Target = 100 mg/dL and AIT = 2 hours | 3 (7) | 4 (9) | 4 (9) |
| Temporary Target | 0 | 1 (2) | 0 |
| 120 mg/dL | 4 (9) | 6 (14) | 6 (14) |
| 110 mg/dL | 7 (16) | 10 (23) | 8 (19) |
| 100 mg/dL | 22 (51) | 26 (60) | 22 (51) |
| Most used AIT (hour: minute) |  |  |  |
| 4:00 | 5 (12) | 6 (14) | 6 (14) |
| 3:30 | 0 | 1 (2) | 1 (2) |
| 3:15 | 1 (2) | 1 (2) | 1 (2) |
| 3:00 | 17 (40) | 21 (49) | 18 (42) |
| 2:30 | 3 (7) | 6 (14) | 3 (7) |
| 2:00 | 7 (16) | 8 (19) | 7 (16) |

**Note**: Data presented as frequency (%).

**Supplementary table 2**. Percent (%) Changes in Device Settings

|  | **Sha'ban** | **Ramadan** | **Shawwal** |
| --- | --- | --- | --- |
| **Most used Target** | | | |
| 120 mg/dL | 12.1 | 14 | 16.7 |
| 110 mg/dL | 21.2 | 23.3 | 22.2 |
| 100 mg/dL | 66.7 | 60.5 | 61.1 |
| **Most used AIT (hour: min)** | | | |
| 4:00 | 15.2 | 14 | 16.7 |
| 3:30 | 0 | 2.3 | 2.8 |
| 3:15 | 3 | 2.3 | 2.8 |
| 3:00 | 51.5 | 48.8 | 50 |
| 2:30 | 9.1 | 14 | 8.3 |
| 2:00 | 21.2 | 18.6 | 19.4 |

**Note**: Data presented as percentage (%).


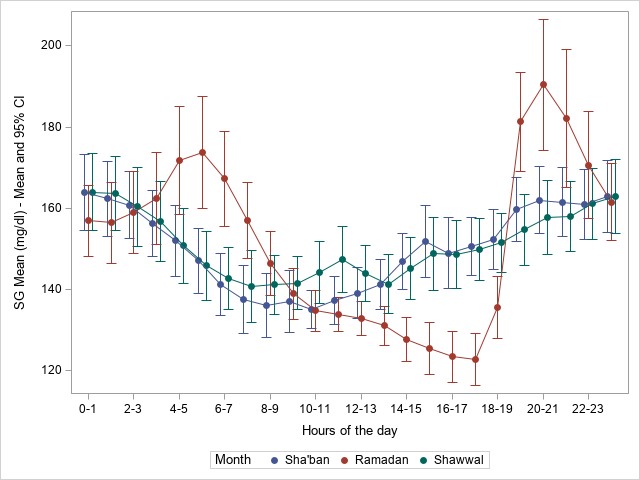


**Supplementary figure 1.** Glucose profiles across months


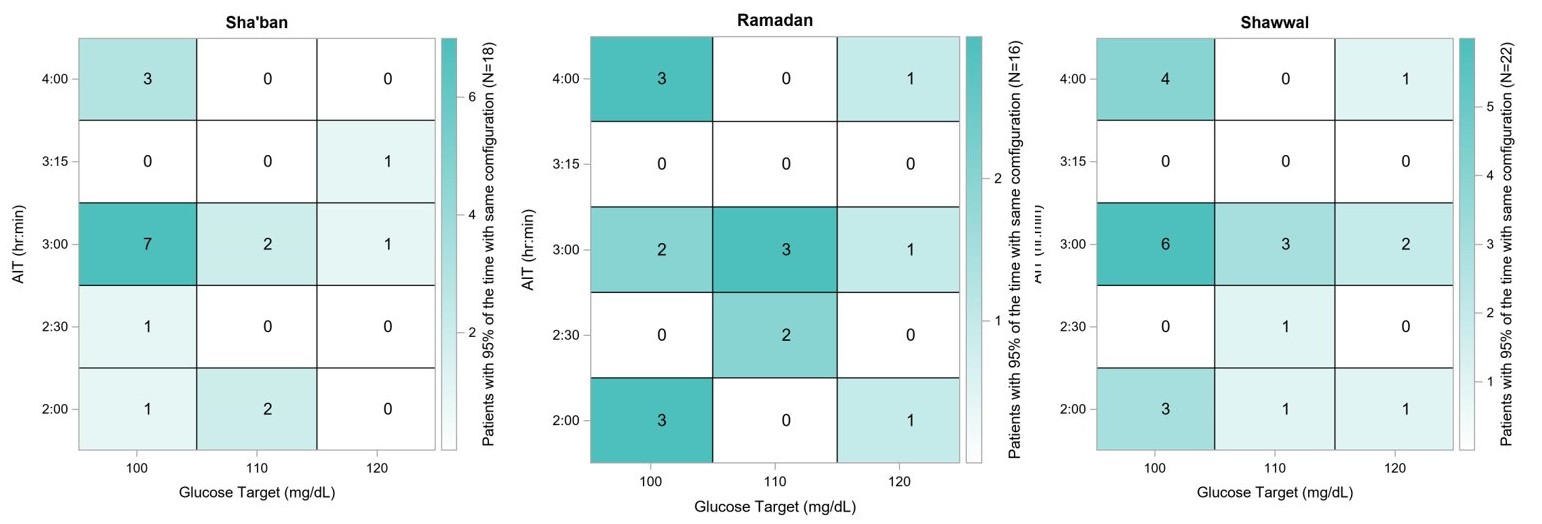


**Supplementary figure 2.** Device configurations used by the patients (for at least 95% of the time) during Sha’ban, Ramadan, and Shawwal.
